# Impact of infection prevention and control training on health facilities during the Ebola virus disease outbreak in Guinea

**DOI:** 10.1186/s12889-018-5444-3

**Published:** 2018-04-24

**Authors:** Mory Keïta, Ansoumane Yassima Camara, Falaye Traoré, Mohamed ElMady Camara, André Kpanamou, Sékou Camara, Aminata Tolno, Bienvenu Houndjo, Fatimatou Diallo, Fatoumata Conté, Lorenzo Subissi

**Affiliations:** 1 Bureau de Pays de l’Organisation Mondiale de la Santé pour la Guinée, Immeuble BAH, Quartier Cameroun, BP: 817, Conakry, Guinea; 2 Public Health Agency of Canada, 130 Colonnade Road, A.L. 6501H, Ottawa, K1A 0K9 Ontario Canada; 3 Université GAN de Conakry - Faculté de Médecine - Pharmacie et Odontostomatologie, Commune de Dixinn, Route de Donka, Quartier Landréah, BP: 1147, Conakry, Guinea; 4 Institut National de Santé Publique, En face du Jardin 2 octobre, BP: 6623, Conakry, Guinea; 5 Ministère de la Santé, Boulevard du commerce - Almamya Kaloum, BP: 585, Conakry, Guinea; 6 Viral Diseases, Scientific Public Health Institute (WIV-ISP), Rue Engeland 642, 1180, Uccle, Bruxelles, Belgium

**Keywords:** Infection control, Infection prevention, Ebola, Guinea, Hospital epidemiology

## Abstract

**Background:**

In 2014–2016, West Africa faced the most deadly Ebola Virus Disease (EVD) outbreak in history. A key strategy to overcome this outbreak was continual staff training in Infection Prevention and Control (IPC), with a focus on Ebola. This research aimed to evaluate the impact of IPC training and the quality of IPC performance in health care facilities of one municipality of Conakry, Guinea.

**Methods:**

This study was conducted in February 2016. All health facilities within Ratoma municipality, Conakry, Guinea, were evaluated based on IPC performance standards developed by the Guinean Ministry of Health. The IPC performance of healthcare facilities was categorised into high or low IPC scores based on the median IPC score of the sample. The Mantel-Haenzsel method and logistic regression were used for statistical analysis.

**Results:**

Twenty-five percent of health centres had one IPC-trained worker, 53% had at least two IPC-trained workers, and 22% of health centres had no IPC-trained workers. An IPC score above median was positively associated with the number of trained staff; health centres with two or more IPC-trained workers were eight times as likely to have an IPC score above median, while those with one IPC-trained worker were four times as likely, compared to centres with no trained workers. Health centres that implemented IPC cascade training to untrained medical staff were five times as likely to have an IPC score above median.

**Conclusions:**

This research highlights the importance of training healthcare staff in IPC and organising regular cascade trainings. IPC strategies implemented during the outbreak should continue to be reinforced for the better health of patients and medical staff, and be considered a key factor in any outbreak response.

**Electronic supplementary material:**

The online version of this article (10.1186/s12889-018-5444-3) contains supplementary material, which is available to authorized users.

## Background

In 2014–2016, the Republics of Guinea, Liberia and Sierra Leone faced the most deadly Ebola Virus Disease (EVD) outbreak in history. WHO declared a Public Health Emergency of International Concern (PHEIC) on August, 8th, 2014, which was lifted on March 29th, 2016. A total of 28,616 confirmed, probable and suspected cases and 11,310 deaths were reported during the outbreak, mostly in Guinea, Liberia and Sierra Leone [1].

**Fig. 1:**
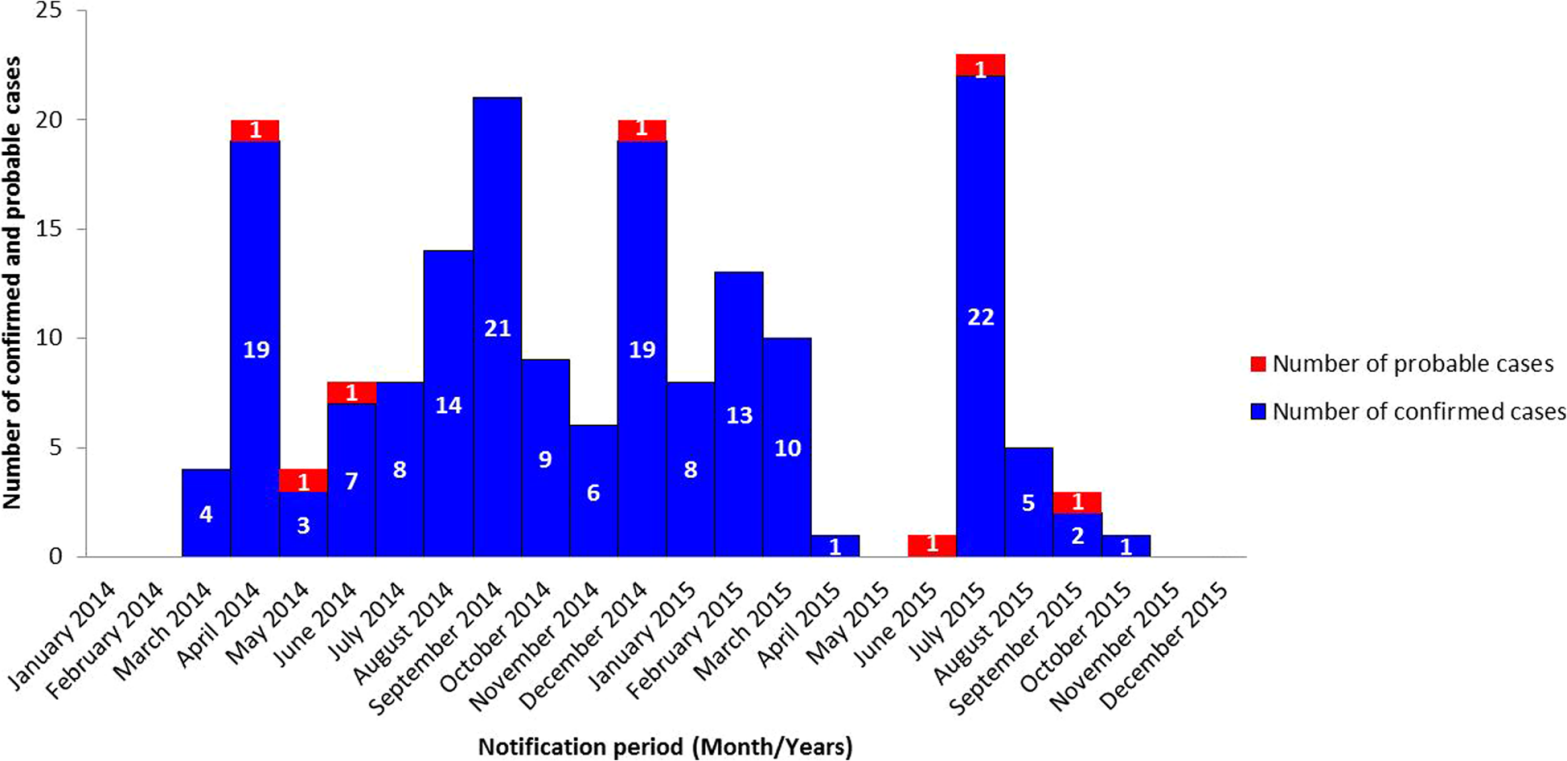
Epidemiological curve by month, Ratoma municipality (Conakry)

In Guinea, there was a total of 3811 probable and confirmed EVD cases, and 2543 deaths [1]. Ratoma, the largest municipality of the capital, Conakry, reported a total of 179 EVD cases (7 probable and 172 confirmed), including 92 deaths (CFR 51.4%). This municipality experienced at least two waves of cases: the second had a peak in July 2015 (22 confirmed cases and 1 probable case) and ended by October 2015 (Fig. 1).

This EVD outbreak highlighted the weaknesses of health care systems across the three countries, including the lack of reliable public health surveillance systems [2]. Prior to the EVD outbreak, Infection Prevention Control (IPC) activities in Liberia were found to be basic: there was no national IPC guidance and no dedicated staff at any level of government or healthcare facility to ensure the implementation of IPC best practices [3]. In Sierra Leone and Guinea, the need for IPC was recognised at all levels of health care delivery as soon as EVD transmission episodes occurred in healthcare facilities [4, 5].

To overcome this unprecedented epidemic, the World Health Organization (WHO) coordinated an international response. The Global Outbreak Alert and Response Network mobilised Operational Support Team including international expertise in clinical case management, surveillance and epidemiology, data management, IPC, outbreak logistics, social mobilisation, risk communications and medical anthropology [6].

Several strategies were implemented to contain and prevent the outbreak to spread to other countries, i.e. prevention through risk and behavioral change, safe case management in Ebola Treatment Units (ETUs), epidemic surveillance by contact tracing, IPC implementation in all health care settings to promote good hand hygiene for patients and health care professionals, safe and dignified burials and implementation of health control at borders. IPC measures included the delivery of personal protective equipment (PPE) (gloves, gowns, mask, and boots) and infection control supplies (chlorine, buckets, disposable rags, soap, etc.). The importance of training and assessing IPC practices in healthcare facilities that were not serving as ETUs was recognised by the US Centres for Disease Control and Prevention (CDC) as a priority [7]. The ‘Ring IPC strategy’ - which consists of providing rapid, intensive and short-term (21-days) support to healthcare facilities and communities in areas of active Ebola transmission - had a good impact in Guinea and Liberia [8, 9].

Throughout the EVD outbreak in Guinea, individual healthcare workers (usually 1 or 2 per healthcare facility) were selected to take part in an intensive five-day IPC training with a focus on EVD, organised by the Ministry of Health and partners (WHO, CDC and others). The participants were strongly encouraged to organise cascade training, i.e. training to other medical staff within their respective healthcare structures, following guidelines developed by the Ministry of Health and as previously described [10].

The purpose of this research was to evaluate healthcare personnel training in healthcare facilities and the subsequent impact on the quality of IPC performance in healthcare facilities within Ratoma municipality.

Two hypotheses were tested in this research:

1. There is a positive association between the health care structure IPC performance and the number of IPC-trained staff.
2. Within an health care facility, IPC cascade training by IPC-trained staff, compared to no cascade training, is more likely to result in higher IPC performance, regardless of the type of health care structure and whether the structure is certified or not (i.e. whether it has government permission to provide health care).

## Methods

This study was conducted in February 2016. All health facilities within Ratoma municipality were evaluated based on the IPC performance standards developed by a team of Guinean medical doctors of the Guinean Ministry of Health who had received training on IPC tools and good practice. All surveyors were Guinean medical doctors and attended a week of intense training on IPC tools and good practices.

IPC performance was evaluated using two observation sheets: the first contained 30 questions corresponding to 30 IPC milestones and was used in all healthcare centers [11]; the second had the same 30 questions, plus three additional questions applicable only to the 4 major hospitals in the municipality (Additional file 1, in French) [12]. Questions included both clinical and non-clinical questions. The non-clinical part indicated if the structure adequately achieved the IPC milestone listed in the observation sheet by marking yes, no, or not applicable. In the same way, the clinical part indicated if the health care structure’s staff adequately performed each task listed in the observation sheet. The overall performance of the facility was calculated by determining the number of IPC milestones achieved by the facility against the number of applicable IPC milestones. In October 2014, twenty-four health facilities spread across the country, including eleven in Conakry, served as pilot sites to test the performance of these IPC evaluation tools.

Written consent was sought from the head of the healthcare facility for participation in this study. Healthcare staff at the facility were informed that study staff evaluating IPC performance were collecting information on other surveillance activities in order to limit observational bias [13]. Certified facilities were those able to show the permission to offer health services signed by the Ministry of Health.

Healthcare facilities were categorised as having either high or low IPC scores by assessing the facility’s IPC score against the median score for this sample. Facilities with a low IPC score comprised facilities that had an IPC score below the median value, while those with a high IPC score had an IPC score of the median value or above. Univariate analysis was conducted for two main exposures (number of trained staff and cascade training) and two confounders (certification status and type of healthcare facility). Variables with a *p*-value < 0,2 in the univariate analysis were retained in the final model of the multivariate analysis. Because we considered them likely to be related, one separate logistic regression model was built for each exposure (number of trained staff and cascade training). The statistical test used for each reported *p*-value is specified below each table. For all analyses, the results were considered significant at the level of uncertainty α = 5% (*p* < 0.05). Models predicted higher IPC scores.

## Results

**Fig. 2:**
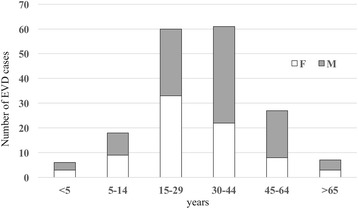
Age and sex distribution of EVD confirmed cases in Ratoma municipality (Conakry)

In Ratoma, the largest municipality of the capital Conakry, the first EVD case was reported in March 2014 and the last one in October 2015 (Fig. 1). Of the EVD cases, 78 were females (44.0%) and 101 were males (56.0%), and the majority of cases were between 15 and 44 years old (Fig. 2). Among health care workers, Ratoma registered 28 confirmed and probable EVD cases including 14 medical doctors, seven nurses, three laboratory workers, two interns, one midwife and one attendant. In Early 2015, the National Sino-Guinean Hospital in Ratoma was among the first health care structures where nosocomial transmission occurred: six health care workers, all contaminated by one patient, died of EVD.

A total of 218 healthcare structures, of which 204 private (94%) and 14 public structures (6%), were included in this study. Among the public structures, one was a national hospital, three were communal hospitals and 10 primary healthcare centers (PHCCs). IPC scores ranged from 10% to 92%, with a median IPC score of 50% (Table 1). All 14 public healthcare structures sector were certified (100%), while 55 private health care structures (27%) were not certified (i.e. had no permission from the Ministry of Health to offer health services).

**Table 1:** Sample description

Variables	Below-median-IPC score	Above-median- IPC score	Total, n (%)	*p*-value*
IPC Score				
Median (range)	–	–	50.0 (10–92)	–
Number of trained staff				
0	40 (36.4)	8 (7.4)	48 (22.0)	< 0.001
1	30 (27.3)	24 (22.2)	54 (24.8)	
2 or more	40 (36.4)	76 (70.4)	116 (53.2)	
Cascade training^a^				
No	46 (41.8)	12 (11.1)	58 (26.6)	< 0.001
Yes	64 (58.2)	96 (88.9)	161 (73.4)	
Type of healthcare facility				
Private	108 (98.2)	96 (88.9)	204 (93.6)	< 0.01
Public	2 (1.8)	12 (11.1)	14 (6.4)	
Certification				
Non-certified	38 (34.5)	17 (15.7)	55 (25.2)	< 0.001
Certified	72 (65.5)	91 (84.3)	163 (74.8)	
Total	110 (100)	108 (100)	218 (100)	

*NS* non-significant

*Chi^2^ test

^a^training for untrained staff by the selected participants of this study

The observation sheet from the vast majority of healthcare facilities revealed common gaps in the sterilization process, inappropriate management of the triage zone, and the lack of a dedicated zone to don and doff PPE.

Twenty-five percent of healthcare structures had one individual externally trained in IPC, 53% had at least two trained individuals, and 22% of healthcare structures did not have any trained individual. Among the structures with no IPC-trained personnel, 7% had an IPC score above the median, while 36% were below the median. Cascade training was implemented in 73% of health centers, and the IPC score was significantly more likely to be above the median when the health center had IPC cascade training (*p* < 0.001). Only two public facilities had an IPC score below median and there was evidence that overall IPC score was significantly more likely to be above the median compared to private facilities (*p* < 0.01). Similarly, certified healthcare structures performed significantly better than non-certified ones (*p* < 0.001).

An IPC score above median was positively associated with the number of IPC-trained staff (Table 2). Health centers with one IPC-trained individual were four times as likely to have an IPC score above median compared to those with no trained staff. Health centers with two or more IPC-trained workers were almost ten times as likely to have an IPC score above median, compared to those with no trained staff. Cascade training was also associated with IPC performance (*p* < 0.001), with health centers that organised a cascade training being approximately six times as likely to have an IPC score above median compared to those that did not. Being a public healthcare facility (compared to being private), and being a certified healthcare facility were also positively associated with IPC score (both *p* < 0.01).

**Table 2:** Univariate Analysis of IPC performance (below- versus above-median-IPC score) and independent variables

Variables	OR (95% CI)	*p*-value*
Cascade training^a^		
No	–	
Yes	5.75 (2.83–11.69)	< 0.001
Number of trained staff		
0	–	
1	4.00 (1.58–10.14)	< 0.01
2 or more	9.50 (4.06–22.23)	< 0.001
Type of healthcare facility		
Private	–	
Public	6.75 (1.47–30.92)	< 0.01
Certification		
Non-certified	–	
Certified	2.83 (1.48–5.41)	< 0.01

Observations with outcome corresponded to health centers with IPC scores above median

*OR* Crude Odds ratio

*Chi^2^ test

^a^training for untrained staff by the selected participants of this study

Multivariate analysis confirmed that the number of trained staff in a healthcare facility was positively associated with IPC score, even after adjusting for type of health facility and certification status (Table 3). Compared to healthcare facilities with no IPC-trained staff, those with one IPC-trained worker were almost four times as likely to have an IPC score above median (aOR 3.85, 95% CI 1.50–9.90), while those with two or more IPC-trained workers were almost eight times as likely to have an IPC score above median (aOR 7.73, 95% CI 3.21–18.64). After adjustment for number of trained staff, facility type and certification status had no effect on IPC performance (Table 3).

**Table 3:** Multivariate analysis of IPC performance (below- versus above-median-IPC score) and the number of IPC-trained staff

Variables	aOR (95% CI)	*p*-value*
Number of Trained staff		
0	–	
1	3.85 (1.50–9.90)	< 0.01
2 or more	7.73 (3.21–18.64)	< 0.001
Type of healthcare facility		
Private	–	
Public	4.48 (0.90–22.22)	NS
Certification		
Non-certified	–	
Certified	1.61 (0.78–3.30)	NS

Observations with outcome corresponded to health centers with IPC scores above median

*aOR* adjusted Odds Ratio, *NS* Non-significant

*Wald test

Finally, when we compared the healthcare facilities that implemented cascade training with those that did not, the first were five times as likely to be ranked among the group with an IPC score above median (Table 4). Again after adjustment, facility type and certification status were not associated with IPC performance (Table 4).

**Table 4:** Multivariate analysis of IPC performance and IPC training cascade training in healthcare facilities

Variables	aOR (95% CI)	*p*-value*
Cascade training^a^		
No	–	
Yes	4.73 (2.28–9.85)	< 0.001
Type of healthcare facility		
Private	–	
Public	1.78 (0.88–3.60)	NS
Certification		
Non-certified	–	
Certified	4.70 (0.98–22.46)	NS

Observations with outcome corresponded to health centers with IPC scores above median

*aOR* adjusted Odds Ratio, *NS*: non-significant

*Wald test

^a^training for untrained staff by the selected participants of this study

## Discussion

This study points at the importance of IPC measures during the EVD outbreak to i) interrupt Ebola virus transmission and ii) protect the health workforce, a population that was disproportionately affected by this EVD outbreak [14].

In a rapid IPC assessment performed at the beginning of the EVD outbreak in Sierra Leone, IPC gaps, including shortages or absence of trained health care staff, PPE, safe patient transport, and standardized IPC protocols, were identified in all six districts [14]. Another study from Sierra Leone highlighted the weak management system of IPC material supplies, poor patient triage, and inadequate IPC training, as well as a lack of monitoring and supervision to support good IPC practice [15].

This EVD outbreak mobilized the international community. The US CDC quickly provided a form highlighting criteria on Ebola prevention and control for US hospitals [16]. The IPC strategy differs whether a hospital is designated or not for EVD patient management, but all hospitals should be able to provide care to any EVD patient for 24 h before referral or assistance from another hospital. In many developed and developing countries, hospital staff was trained in the identification of suspected cases to quickly put in place IPC measures aiming at protecting other patients, visitors and health workers. This included the use of PPE, which greatly reduces the contamination risk. In our study, the score given to evaluate the PPE procedure was based on the observation of only one or two health workers of the health care center and we cannot be confident that the observed staff members were free from Hawthorne effects [13].

Observational studies of IPC practices may fail to provide a true reflection of standard IPC practices in a healthcare facilities due to staff systematically altering their behaviour when they know that they are being observed [13]. This research used a blind strategy, i.e. workers were blinded to the fact that their IPC practices were being assessed. According to the gaps evidenced in each observation sheet, appropriate IPC recommendations were given to each visited healthcare center.

The EVD outbreak mobilized both private and public healthcare facilities, regardless of their certification status. It is therefore not surprising to observe that i) training staff in IPC and ii) cascade training across different healthcare facilities played a more significant role than a facility’s public or private nature or certification status. This study did observe higher IPC scores in public healthcare facilities compared to private ones (Table 1): this is possibly due to better financial support from the government and national and international non-governmental organizations during the EVD outbreak, when additional funding became rapidly available. Despite this possibility, non-certified private services were regularly assisted and monitored for their IPC performance by the government and the Ebola national coordination,

The main limitation of our study was that our analysis did not allow to show which variable between cascade training and number of trained staff was the most important in this setting. This could have been helpful for health centers allocating limited resources during future outbreaks. Another limitation is that we did not collect other variables that may affect IPC control, such as size of the healthcare facility or proportion of clinicians among hospital staff.

This research focused on health care centers, and the community was not the target of this IPC evaluation. However, adherence of the community to IPC precautions was key to overcoming this EVD outbreak. Marais et al. recognised that while rigorous adherence to standard IPC precautions and safety standards for Ebola in the community is critical, outbreak control is likely to be even more successful when local community knowledge and experiences of IPC medical teams are combined [17].

A programme against non-certified healthcare facilities was launched in January 2016 with the aim of improving patients’ security and health. As a consequence of this, soon after this research many non-certified clinics stopped providing services. Regular assessments of IPC performance in healthcare facilities are essential activities to identify health services of low quality and should be integrated within the national health programme.

## Conclusion

This research highlights the need for services in charge of healthcare management during an outbreak to train staff in IPC and organise regular cascade trainings. During an outbreak, healthcare workers fear contracting the disease and healthcare facilities fear stigmatization and closure of their services. Better IPC management leads to significantly improved performance among private and public healthcare services, helping to prevent nosocomial infections. IPC strategies implemented during the EVD outbreak should be kept and reinforced for the better health of patients and healthcare workers.

## Additional file

Additional file 1: Standards performance assessment tool for infection prevention and control, used in Guinea during the Ebola virus disease outbreak, 2014–2016. (PDF 438 kb)

## Abbreviations

95% CI: 95% Confidence Interval
aOR: Adjusted odds ratio
CFR: Case fatality ratio
EVD: Ebola Virus Disease
IPC: Infection Prevention Control
OR: Crude odds ratio
PHCC: Primary health care centre
PPE: Personal protective equipment
