# Supplementary material for: Impact of infection prevention and control training on health facilities during the Ebola virus disease outbreak in Guinea
Source: BMC Public Health. 2018 Apr 24;18:547. doi: 10.1186/s12889-018-5444-3 (PMC5921271; doi:10.1186/s12889-018-5444-3)
Supplement: Supplementary file 1 — Standards performance assessment tool for infection prevention and control, used in Guinea during the Ebola virus disease outbreak, 2014–2016. (PDF 438 kb) [file 12889_2018_5444_MOESM1_ESM.pdf]

STANDARDS DE PERFORMANCE POUR LES MESURES DE PREVENTION DES INFECTIONS
MINISTERE DE LA SANTÉ ET DE L'HYGIÈNE PUBLIQUE
REPUBLIQUE DE GUINÉE

STRUCTURE SANITAIRE :____

EVALUATEUR :________________________________ **DATE:** __________________

Instructions:

Pour les domaines non cliniques, indiquez si l'administration a assumé de manière adéquate les tâches consignées dans cette fiche d'observation qui lui sont assignées en marquant un «O» (oui). Marquez «N» si non, et « NA » si non applicable.

Pour les domaines cliniques, indiquer :

- Si le prestataire a effectué de manière adéquate chaque tâche de la fiche d'observation veillez marquer un «O».
- Si le prestataire a effectué la tâche incorrectement ou s'il ne l'a pas effectuée veuillez marquer un « N»
- Si l'une des tâches n'est pas applicable, marquez un «N/A»

Chaque standard est atteint lorsque 100% des critères applicables sont observés

Prière d'écrire également des remarques supplémentaires concernant la performance du prestataire dans la colonne "Commentaires".

L'observateur doit éviter d'intervenir au cours de l'utilisation de cette fiche. Il ne s'agit pas d'un encadrement.

STANDARDS DE PERFORMANCE	CRITERES DE VERIFICATION	[1]O, N ou N/A	COMMENTAIRES
**1** La structure sanitaire est propre	Vérifier, lors de la visite, l'**absence** de poussière, de sang, de déchets, d'aiguilles et de seringues utilisées et de toiles d'araignée dans les endroits suivants :<br>• Terrain externe (en dehors de la structure))<br>• Salle d'admission/d'attente/de tri/d'isolement/d'examen<br>• Salles des soins<br>• Salle de consultation prénatale<br>• Pharmacie<br>• Laboratoire<br>• Banque de sang	____<br>____<br>____<br>____<br>____<br>____<br>____<br>____	

[1] O = Oui; N = Non; N/A = Non Applicable

STANDARDS DE PERFORMANCE	CRITERES DE VERIFICATION	[1]O, N ou N/A	COMMENTAIRES
	• Salle de travail<br>• Salle d'accouchement<br>• Zone du post-partum immédiat<br>• Zone pour les soins immédiats du nouveau-né<br>• Salle d'opération<br>• Salle de PF<br>• Zone de traitement des instruments<br>• Zone de stockage<br>• Salle d'hospitalisation<br>• Salle de réveil<br>• Lavabos et toilettes<br>• Tables d'examen<br>• Salle d'échographie<br>• Salle d'endoscopie<br>• Salle de radiologie<br>• Salle de scanner<br>• Salle de réunion/staff<br>• Couloir<br>• Comptoir<br>• Etagères<br>• Chaises<br>• Eviers	________<br>________	

STANDARDS DE PERFORMANCE	CRITERES DE VERIFICATION	O N NA	COMMENTAIRES
**2** La structure Sanitaire dispose d'un point d'eau propre dans chaque unité de prestation de service<br><br>*(Bassin pour le lavage des mains équipé de robinet à pédale, à main ou muni d'un Gobelet)*	Observer s'il y a de l'eau propre dans les endroits suivants :<br>• Salle de Tri<br>• Salle d'examen<br>• Salle de conseils/consultation<br>• Salles des soins prénatals<br>• Salle d'observation / hospitalisation.<br>• Laboratoire<br>• Banque de sang<br>• Salle de travail<br>• Salle d'accouchement<br>• Zone du post-partum immédiat<br>• Zone pour les soins immédiats du nouveau-né<br>• Salle d'opération<br>• Salle de PF<br>• Zone de traitement des instruments<br>• Salle d'hospitalisation<br>• Salle de réveil<br>• Salle d'échographie<br>• Salle d'endoscopie<br>• Salle de radiologie<br>• Salle de scanner<br>• Salle de réunion/staff	______<br>______	

STANDARDS DE PERFORMANCE	CRITERES DE VERIFICATION	O N NA	COMMENTAIRES
**3**. La formation sanitaire dispose de matériels et de consommables nécessaires a l'entretien en quantité suffisante pour au moins un mois	Vérifier lors de la visite la disponibilité de :<br>• gants de ménage<br>• serpillères<br>• tabliers / blouses jetables ou réutilisables<br>• bottes<br>• bavettes<br>• lunettes/ masque pour le visage/ écran facial/visière<br>• Ballais<br>• Raclettes<br>• Seaux<br>• Seaux avec robinet<br>• Pulvérisateurs	 ______<br>______<br>______<br>______<br>______<br>______<br>______<br>______<br>______<br>______<br>______	
**4.** La structure sanitaire dispose d'antiseptiques et de désinfectants pour au moins un mois.	Vérifier, dans la salle d'entreposage et/ou de stockage, en comparaison à l'utilisation des mois précédents, tel qu'indiqué sur le formulaire de contrôle de stock, l'existence et les quantités des matériaux consommables suivants :<br>**Antiseptiques :**<br>• Chlorhexidine (solution)<br>• Alcool, 60–90% (alcool dénaturé)<br>**Désinfectants :**<br>• Hypochlorite de sodium (eau de Javel)<br>• Ethyle, alcool isopropylique (60%–90%), **ou**<br>• Cetrimide et chlorhexidine gluconate (2%–4%) (Savlon®), **ou**<br>• Chlorhexidine gluconate (2%–4%) (Hibiclens®, Hibiscrub®, Hibitane®), **ou**<br>• Préparations iodées (1%–3%) par exemple, Lugol, **ou**<br>• Iodophore (1:2500; par exemple, Bétadine®)<br>• Savon/détergent	______<br>______<br>______<br>______<br>______<br>______<br>______<br>______<br>______	

**STANDARDS DE PERFORMANCE**	**CRITERES DE VERIFICATION**	**O N NA**	**COMMENTAIRES**
**5** La concentration et l'utilisation des antiseptiques pour préparer la peau et / ou les muqueuses est respectée	Observer, dans chacune des zones de prestations de services :<br>**Si :**<br>• La concentration antiseptique est étiquetée avec le nom et la concentration tel que suit :<br>– Ethyle, alcool isopropylique (60%–90%), **ou**<br>– Cetrimide et chlorhexidine gluconate (2%–4%) (Savlon®), **ou**<br>– Chlorhexidine gluconate (2%–4%) (Hibiclens®, Hibiscrub®, Hibitane®), **ou**<br>– Préparations iodées (1%–3%) par exemple, Lugol, **ou**<br>– Iodophore (1:2500; par exemple, Bétadine®), ou<br>– Parachlorométaxylénol ou PCMX (0,5-4%) : Dettol<br>• Les antiseptiques sont préparés dans de petits récipients réutilisables pour utilisation quotidienne<br>• Les récipients réutilisables sont soigneusement lavés avec de l'eau et du savon, rincés avec de l'eau propre et séchés avant d'être remplis à nouveau<br>• Les récipients réutilisables sont étiquetés avec la date à chaque fois qu'ils sont remplis à nouveau<br>• La gaze ou le coton sont stockés dans des récipients secs sans antiseptique<br>• Les instruments et autres articles sont stockés dans des récipients secs sans antiseptiques	______<br>______<br>______<br>______<br>______<br>______<br>______<br>______<br>______<br>______<br>______	

STANDARDS DE PERFORMANCE	CRITERES DE VERIFICATION	O N NA	COMMENTAIRES
**6** La décontamination des instruments et autres objets (immédiatement après l'utilisation et avant le nettoyage) est réalisée correctement.<br>**Formule de dilution eau de javel :**<br>$\text{Nbre mesure d'eau} = \left[\frac{\text{\% Conc. Disponible}}{\text{\% Conc. Souhaitée}}\right] - 1$<br>Nb : si la conc. Disponible est en degré, il faut transformer en % en tenant compte qu'un degré égal à 0,3%.<br><br>Formule de dilution Hypochlorite (poudre) :<br>$\text{Grammes/litre} = \left[\frac{\text{\% Conc. Souhaitée}}{\text{\% Conc. Disponible}}\right] \text{X } 1000$<br><br>**Exemples** : La concentration de la solution de chlore est de 0,5% (Conc. Souhaitée):<br>- **Hypochlorite de sodium liquide (Javel) :** (3,5%), 1 part de Javel pour 6 parts d'eau, **ou**<br>**Hypochlorite de calcium (poudre) :** (35%), 14 grammes de poudre pour 1 litre d'eau.	Observer si :<br>• La formule de dilution est affichée dans toutes les salles de procédures<br>• La solution de décontamination est disponible dans toutes les salles de procédures<br>• Vérifier en se référant aux formules de dilution si la préparation de la solution de décontamination est correcte en se basant sur la concentration de base du chlore disponible (Javel ou hypochlorite)<br>• La formation sanitaire prépare une nouvelle solution de chlore au début de chaque jour ou plus souvent, si nécessaire<br>• On immerge les instruments et autres objets dans la solution de chlore à 0,5% pendant 10 minutes.<br>• La formation sanitaire utilise des récipients avec une solution chlorée propre à 0,5% pour chaque intervention chirurgicale et change la solution après la procédure.<br>• Après 10 minutes, les instruments et autres objets sont retirés de la solution chlorée et rincés avec de l'eau propre ou nettoyés immédiatement.	______<br>______<br>______<br>______<br>______<br>______<br>______<br>______	

**7** Il existe une zone séparée pour le traitement des instruments souillés	Observer, dans la structure sanitaire :<br>**Si :**<br>• la zone de traitement des instruments est séparée des zones d'intervention<br>• Les articles sales ne sont pas au contact des articles propres<br>• Il existe au moins un évier avec l'eau courante/point d'eau pour laver les instruments<br><br>**Si les instruments sont enveloppés/emballés dans cette zone :**<br>• Il existe un plan de travail propre pour envelopper/emballer les instruments disposant de :<br>– Une table de travail<br>– Des étagères pour ranger les paquets propres<br>– Un bureau pour la tenue de dossiers<br>• Les instruments enveloppés/emballés sont envoyés pour la stérilisation	______<br>______<br>______<br><br>______<br>______<br>______<br>______	

STANDARDS DE PERFORMANCE	CRITERES DE VERIFICATION	O N NA	COMMENTAIRES
**8** le processus de nettoyage des instruments et autres articles est réalisé correctement<br><br>.	Observer si la personne qui nettoie les instruments le fait conformément aux étapes et recommandations standards :<br>• Porte :<br>- gants de ménage<br>- couverture de la tête<br>- masque, lunettes ou écran facial<br>- tablier en plastique<br>- chaussures fermées/bottes<br>• Utilise :<br>- une brosse souple<br>- détergeant<br>- eau courante<br>• Frotte les instruments et autres objets sous l'eau, enlevant complètement tout le sang et autre matière étrangère	 <br>______<br>______<br>______<br>______<br>______<br>______<br><br>______<br>______<br>______<br>______<br><br>______	

STANDARDS DE PERFORMANCE	CRITERES DE VERIFICATION	O N NA	COMMENTAIRES
	• Démonte les instruments et autres objets ayant de multiples parties, les nettoie avec une brosse, notamment les cannelures, dents et jointures.<br>• Rince les instruments et autres objets soigneusement avec de l'eau propre<br>• S'assure que le matériel est visiblement propre<br>• Laisse sécher les instruments et autres objets à l'air ou les sèche avec une serviette propre<br>• Se lave les mains après avoir retiré les gants	______<br>______<br>______<br>______<br>______	
**9** La collecte et le traitement de linges souillés sont réalisés correctement pour éviter les blessures et la contamination	Observer, dans chacune des zones de prestation de services si :<br>• On collecte les linges souillées dans des récipients étanches /sachets en plastiques sans qu'ils aient été trempés ou lavés dans la salle<br>• Le personnel de nettoyage porte des gants ménagers lorsqu'ils ramassent les linges souillés<br>• Les linges sont envoyés à la laverie dans des conteneurs fermés (seaux, sacs en plastique ou chariots) pour être triés, lavés et séchés	______<br>______<br>______<br>______	
9a. Les dispositions appropriées sont prises au niveau de la Buanderie/Laverie pour la PI	Observer si dans ce service les agents :<br>• Utilisent l'EPI (bottes, Gants de ménage, tablier, lunette)<br>• Vérifient que le linge souillé est préalablement Décontaminé si nécessaire ou a été passé au prélavage au cas échéant, procèdent à la décontamination/prelavage avant de procéder au lavage<br>• Veillent à la séparation des linges lavés des autres linges souillés. Veillent à ce que le linge ne soit pas mis sur le sol pour être séché.<br>• Les linges lavés et séchés sont transportés pour la stérilisation dans des conteneurs fermés, lavés et décontaminés au préalable	______<br>______<br>______<br>______	

STANDARDS DE PERFORMANCE	CRITERES DE VERIFICATION	O N NA	COMMENTAIRES
**10** le processus d'emballage des objets et matériels à stériliser est correctement fait et selon le type de stérilisation<br>.	Observer lors de l'emballage, si :<br>• Les instruments sont propres et secs<br>**Emballage pour la stérilisation à la vapeur (autoclave**)<br>• Les articles en tissu ont été lavés, séchés et sont intacts (**sans** trous)<br>• Tous les instruments avec joints **sont ouverts ou se trouvent en position non fermée**<br>• Tous les instruments sont démontés<br>**Les types de matériaux utilisés pour emballage sont les suivant**<br>• Emballages en tissu, mousseline ou coton : double emballage<br>• Papier (Kraft ou autre papier) : double emballage<br>• Si on utilise le papier on ne l'utilise qu'une fois et on le jette<br>**Emballage pour la stérilisation à la chaleur sèche :**<br>**Les types de matériaux utilisés sont les suivants** :<br>– Emballages en tissu, mousseline : double emballage<br>– Aluminium, ou<br>– Récipients en métal avec couvercles	______<br>______<br>______<br>______<br>______<br>______<br>______<br>______<br>______<br>______	
**11**. Le processus pour charger le stérilisateur est réalisé correctement selon le type du chargement et du stérilisateur.	**Stérilisation à la vapeur (autoclave):**<br>Observer lors du chargement si :<br>• On laisse au moins 7–8 cm d'espace entre les paquets et les parois de la chambre de stérilisation.<br>• Les paquets en tissus sont rangés sur le côté.<br>• Les récipients en métal solide et en verre sont placés sur le côté avec les fermetures en place	______<br>______<br>______	

STANDARDS DE PERFORMANCE	CRITERES DE VERIFICATION	O N NA	COMMENTAIRES
	• Les plateaux pour instruments (fond grillé ou perforé seulement) sont placés à plat sur les étagères.<br>• Les gants sont stérilisés à part et sont placés sur les étagères supérieures.<br>• Le stérilisateur n'est pas surchargé :<br>- Les paquets et les récipients ne sont pas entassés les uns contre les autres.<br>- La taille des paquets : dimensions maximales : 30x 30x 50 cm ou 5kg<br>- Il y a un espace entre les paquets et les récipients<br>**Stérilisation par chaleur sèche** :<br>Observer lors du chargement si :<br>• On laisse au moins 7–8 cm d'espace entre les paquets et les parois de la chambre de stérilisation.<br>• Le stérilisateur n'est pas surchargé ; les paquets et les récipients ne sont pas entassés les uns contre les autres.	______<br>______<br>______<br>______<br>______<br>______<br>______	
**12**. le processus de stérilisation est correctement réalisé selon le type de stérilisation	Observer lors du cycle de stérilisation si les conditions standard ci-dessous pour les divers modes de stérilisation sont respectées:<br>**Stérilisation à la vapeur (autoclave)**:<br>• 20 minutes pour les articles non emballés ou 30 minutes pour les articles emballés, à 121 ºC (250 ºF) 106 kPa (15 lbs/in²) dans un stérilisateur à déplacement par gravité, **et/ou**<br>• Autre, selon le type d'articles, s'il est emballé ou non, et le type de stérilisateur (selon les instructions du fabricant).<br>**Stérilisation par chaleur sèche**<br>• 170 ºC (340 ºF) pendant une heure après avoir atteint la température souhaitée (cycle total entre 2 heures et 2,5 heures), **et/o**u	_____<br>______<br>______	

STANDARDS DE PERFORMANCE	CRITERES DE VERIFICATION	O N NA	COMMENTAIRES
	• 160 ºC (320 ºF) pendant 2 heures après avoir atteint la température souhaitée (cycle total entre 3 heures et 3,5 heures).<br>**Stérilisation chimique**:<br>• Les instruments démontés sont trempés complètement pendant au moins 10 heures dans une solution de glutaraldéhyde à 2%–4% ou au moins pendant 24 heures dans une solution de formaldéhyde à 8%<br>• Après 10 heures ou 24Heures, les instruments sont retirés avec des gants stériles ou une pince et rincés dans de l'eau stérile, séchés et placés dans un récipient stérile.<br>• Le récipient porte une étiquette indiquant le temps du commencement de la stérilisation.<br>• Le récipient porte une étiquette indiquant la date de reconstitution, et la solution est utilisée dans les 14 jours	______<br>______<br>______<br>______<br>______	
**13** Le processus de décharger le stérilisateur est réalisé correctement selon le type du chargement et du stérilisateur.	**Stérilisation à la vapeur (autoclave)**:<br>Observer lors du déchargement si :<br>• La porte du stérilisateur est ouverte de 12 à 14 cm après la fin du cycle de stérilisation et que la jauge de pression dans la chambre de stérilisation est revenue à « 0 »<br>• On attend 30 minutes avant de décharger le stérilisateur, pour laisser sécher les paquets et les instruments.<br>• Si l'on utilise un chariot de déchargement, le chariot est éloigné du stérilisateur, de la fenêtre ouverte ou de l'éventail électrique jusqu'à ce que le contenu soit refroidi.<br>• Si l'on n'utilise pas de chariot, les paquets sont étalés sur une surface matelassée avec du papier ou du tissu, loin des fenêtres ouvertes ou d'un éventail, jusqu'à ce qu'ils soient refroidis.	______<br>______<br>______<br>______	

STANDARDS DE PERFORMANCE	CRITERES DE VERIFICATION	O N NA	COMMENTAIRES
	• On évite de manipuler les paquets inutilement.<br>• Lorsque les paquets ont refroidi à la température ambiante, on les utilise ou les place dans la zone de stockage.<br>**Stérilisation à la chaleur sèche :**<br>• Les paquets sont étalés sur une surface matelassée propre avec du papier ou du tissu, loin des fenêtres ouvertes ou d'un éventail, jusqu'à ce qu'ils soient refroidis<br>• Les paquets refroidissent à la température ambiante de la salle avant d'être manipulés.<br>• On évite de manipuler les paquets inutilement.<br>Lorsque les paquets /récipients sont refroidi à la température ambiante, on les utilise ou les place dans la zone de stockage.	______<br>______<br>______<br>______<br>______	
**14** La formation sanitaire dispose d'un système pour suivre l'efficacité de la stérilisation en utilisant des indicateurs mécaniques, ou chimiques selon le type de stérilisation	Vérifier, dans la salle de stérilisation si:<br>• des rubans ou étiquettes, qui notent l'heure, la température et la pression, sont utilisés à l'intérieur et à l'extérieur de chaque paquet ou récipient<br>• Il existe un registre pour noter la date, l'heure, la température et la pression pour chaque chargement<br>• Le registre est rempli et revu après chaque chargement<br>• Il y a le nom et la signature de la personne responsable de chaque chargement	_____<br>_____<br>_____<br>_____	

STANDARDS DE PERFORMANCE	CRITERES DE VERIFICATION	O N NA	COMMENTAIRES
**15** La désinfection de haut niveau (DHN) est réalisée correctement en fonction de la méthode utilisée.	Observer pendant le cycle de DHN si les conditions standard ci-dessous sont suivies :<br>• La formation sanitaire dispose d'une minuterie fonctionnelle<br>**Pour l'ébullition** :<br>• Les instruments propres et démontés sont complètement immergés dans l'eau.<br>• Le récipient est recouvert.<br>• Les instruments sont bouillis pendant 20 minutes, chronométrées depuis le moment où commence l'ébullition à gros bouillons.<br>• On **n'ajoute aucun** instrument une fois que l'on a commencé à compter.<br>• Après 20 minutes, les instruments sont retirés avec une pince désinfectée à haut niveau ou avec une pince ou des gants stériles, séchés et entreposés dans des récipients désinfectés à haut niveau.<br>**Pour la DHN à la vapeur :**<br>• La quantité d'eau dans le récipient inférieur de la marmite suffit pour le cycle de vapeur intégral.<br>• Les instruments nettoyés et démontés sont dans les plateaux supérieurs.<br>• La marmite est recouverte.<br>• Les instruments sont traités à la vapeur pendant 20 minutes, commençant à compter au moment où la vapeur commence à sortir entre le plateau et le couvercle.<br>• On **n'ajoute aucun** instrument une fois que l'on a commencé à compter.	______<br>______<br>______<br>______<br>______<br>______<br>______<br>______<br>______<br>______<br>______	

STANDARDS DE PERFORMANCE	CRITERES DE VERIFICATION	O N NA	COMMENTAIRES
	• Après 20 minutes, les instruments sèchent à l'air ambiant et sont entreposés dans les plateaux couverts ou ils sont retirés avec une pince désinfectée à haut niveau ou une pince ou des gants stériles, séchés et entreposés dans des récipients désinfectés à haut niveau.<br>**Pour la DHN chimique :**<br>• On prépare le glutaraldéhyde (en concentration selon les instructions du fabricant) ou une solution de chlore à 0,1% avec de l'eau bouillie:<br>• Les instruments nettoyés et démontés sont immergés dans la solution de glutaraldéhyde ou de chlore à 0,1% pendant 20 minutes dans un récipient avec un couvercle.<br>• Le récipient porte une étiquette indiquant le temps du commencement de la DHN.<br>• Après 20 minutes, les instruments sont retirés avec une pince ou des gants désinfectés à haut niveau ou stériles et rincés dans de l'eau stérile ou bouillie, séchés et placés dans des récipients désinfectés à haut niveau.<br>• Le récipient porte une étiquette indiquant la date de reconstitution, et la solution est utilisée dans les 14 jours, si l'on utilise le glutaraldéhyde ou dans les 24 heures si l'on utilise une solution chlorée	______<br>______<br>______<br>______<br>______	
**16** Le processus de stockage des objets stériles ou désinfectés à haut niveau est correctement réalisé.	Observer si :<br>• Les fournitures propres **ne sont pas** entreposées avec les articles stériles ou désinfectés à haut niveau.<br>• Les articles non emballés DHN sont utilisés immédiatement ou entreposés dans des récipients DHN.<br>• Les paquets désinfectés à haut niveau et/ou les récipients sont marqués avec la date d'expiration	______<br>______<br>______	

STANDARDS DE PERFORMANCE	CRITERES DE VERIFICATION	O N NA	COMMENTAIRES
	• Les paquets stériles et/ou les récipients sont marqués avec la date d'expiration.<br>• Les paquets stockés sont :<br>- secs<br>- intacts (sans déchirures ou trous)<br>• Il existe un système de rotation et d'inventaire pour surveiller l'utilisation des articles stérile ou DHN	______<br>______<br>______<br>______	
**17** La solution désinfectante pour le nettoyage est préparée correctement.	Vérifier si la solution désinfectante pour le nettoyage est préparée de la manière suivante :<br>• Une solution chlorée à 0,5% est préparée.<br>• Un détergent (ne contenant pas d'acide, d'ammonium ou de chlorure d'ammonium) est ajouté à la solution chlorée à 0,5% pour obtenir une solution de nettoyage douce et savonneuse.	______<br>______	
**18** Les tables de procédures et les surfaces sont correctement nettoyées	Vérifier qu'après chaque procédure les surfaces suivantes sont essuyées avec une solution chlorée à 0,5% et sont nettoyées avec une solution de nettoyage au moins une fois par mois:<br>• les paillasses de la salle d'accouchement, du laboratoire de la pharmacie<br>• la table d'accouchement<br>• la table de procédure AMIU<br>• la table de petite chirurgie<br>• la table de bloc opératoire<br>• les tables de consultation<br>• les tables de soins<br>• les plaques chauffantes<br>• les pèse bébé<br>• les lits d'hospitalisation<br>• les planchers	_____<br>_____<br>_____<br>_____<br>______<br>_____<br>______<br>______<br>______<br>______	

STANDARDS DE PERFORMANCE	CRITERES DE VERIFICATION	O N NA	COMMENTAIRES
	• les tables de chevets<br>• les lavabos et éviers	______	
**19** L'équipement de nettoyage est décontaminé, nettoyé et séché avant d'être réutilisé ou entreposé.	Observer si les serpillières, seaux, brosses et chiffons de nettoyage sont :<br>• décontaminés en les trempant pendant 10 minutes dans une solution chlorée à 0,5% ou un autre désinfectant adapté.<br>• lavés dans de l'eau et un détergeant.<br>• rincés à l'eau propre<br>• complètement séchés avant d'être réutilisés	______<br>______<br>______<br>______	
**20** La gestion des déchets médicaux est correctement effectuée.	Observer dans la formation sanitaire si :<br>• Il existe suffisamment de poubelles à l'extérieur de l'établissement de santé (sur le site) pour les déchets généraux pour éviter de les jeter par terre ;<br>• Le personnel d'entretien porte l'équipement protecteur pour manipuler les déchets médicaux :<br>- Gants de ménage<br>- Chaussures fermées<br>• Les déchets médicaux sont ramassés de la zone de soins des clients et transportés dans des récipients fermés vers le dépôt provisoire pour évacuation :<br>- Les déchets contaminés liquides sont versés dans un évier, dans des toilettes à chasse d'eau ou dans des latrines à fosse.<br>- Les objets tranchants et piquants sont évacués dans des récipients solides qu'on ne peut pas perforer (carton épais, plastique dur ou récipients métalliques).<br>- Autres déchets médicaux (par exemple, boules de coton utilisées, gaze, pansements, etc.) sont évacués dans des récipients étanches couverts.	______<br>______<br>______<br>______<br>______<br>______	

STANDARDS DE PERFORMANCE	CRITERES DE VERIFICATION	O N NA	COMMENTAIRES
	- Les déchets généraux sont ramassés de toutes les zones dans des récipients couverts bien ajustés<br>- On ferme les récipients et les ramasse lorsqu'ils sont aux trois quarts pleins.<br>• Le personnel d'entretien effectue l'hygiène des mains après avoir manipulé les déchets et retiré les gants de ménage :<br>- Se lave les mains avec l'eau courante et du savon pendant 10 à 15 secondes et les sèche avec une serviette individuelle propre ou une serviette en papier ou les laisse sécher à l'air ambiant, **ou**<br>- Frotte les mains avec 3 à 5 ml d'une solution alcoolisée jusqu'à ce qu'elles soient sèches (si les mains ne sont pas visiblement souillées)	______<br>______<br>______<br>______	
**21**. La structure sanitaire dispose d'un incinérateur ou d'une fosse à déchet fonctionnels pour l'élimination finale des déchets	Vérifier :<br>**Si les déchets sont incinérés :**<br>• La circulation à cet endroit est régulée et accessible uniquement par le personnel en charge<br>• Lors de l'incinération, il y a des flammes<br>• Les cendres des produits incinérés sont jetées comme déchets non contaminés<br>• Il n'y a pas de déchets sur le terrain aux alentours<br>**Si les déchets sont enterrés dans une fosse :**<br>• L'endroit n'est pas accessible pour les autres membres du personnel, la communauté et les animaux domestiques<br>• Le site où les produits sont enterrés est revêtu d'un matériau à faible perméabilité (par exemple, de la terre battue)<br>• Le site où les produits sont enterrés se situe à au moins 50 mètres de tout point d'eau et dans un endroit qui ne risque pas d'être inondé	______<br>______<br>______<br>______<br>______<br>______<br>______	

STANDARDS DE PERFORMANCE	CRITERES DE VERIFICATION	O N NA	COMMENTAIRES
	• La fosse mesure environ 1 mètre carré et 2 mètres de profondeur<br>• Les déchets que l'on a jetés sont recouverts de 10–15 cm de terre tous les jours<br>• La couche de terre finale est de 50–60 cm<br>• La fosse où les produits sont enterrés ne sert pas pendant plus de 30–60 jours maximum<br>• Il n'y a pas de déchets sur le terrain aux alentours	______<br>______<br>______<br>______<br>______	

Organisation du service de PCI - Ebola

STANDARDS DE PERFORMANCE	CRITERES DE VERIFICATION	[2]O, N ou N/A	COMMENTAIRES
**22.** les **directives de prévention et contrôle des infections** (PCI) sont disponibles (affichées et visibles)	Observer si,<br>• Des Précautions standard de PCI existent (document directives, protocoles)		
	• Des Procédures standards pour la demande d'ambulance existent / affichées		
	• Les Procédures standards pour l'enlèvement de cadavre existent / affichées		
	• Les Procédures standards pour documenter / faire des rapports / gérer le personnel suspects ayant participé à des événements qui les expose à l'infection sont disponibles		
	• Le dépistage quotidien de tout le personnel en arrivant au travail, y compris le contrôle de la température, se fait régulièrement en période de la MVE		
	• L'instruction ou directive sur l'interdiction du personnel malade de venir au travail, existe et appliqué ? (Demander : Depuis la dernière vérification y-a-t-il eu un personnel malade venu au travail) en période de la MVE		

[2] O = Oui; N = Non; N/A = Non Applicable

**STANDARDS DE PERFORMANCE**	**CRITERES DE VERIFICATION**	**[3]O, N ou N/A**	**COMMENTAIRES**
**23. Zone de tri** aménagée et gérée de façon adéquate	Observer si,<br>• Le Dispositif de lavage des mains pour les patients et visiteurs est disponible		
	• Tous les patients sont examinés par rapport à la maladie à virus Ébola		
	• Il y a un agent désigné pour la zone de tri (spécifier les heures de travail)		
	• L'EPI est disponible et accessible pour l'agent de tri		
	• Les recommandations pour la gestion des déchets et l'hygiène des mains sont mises en place		
	• Les Patients sont assis au moins à 2 mètres de distance du dépisteur / ou d'autres patients pendant le processus de tri		

[3] O = Oui; N = Non; N/A = Non Applicable

STANDARDS DE PERFORMANCE	CRITERES DE VERIFICATION	[4]O, N ou N/A	COMMENTAIRES
**24 Une Zone d'isolement des patients suspects** est aménagée et gérée adéquatement	Vérifier si,<br>• Une ou plusieurs zones d'isolements sont aménagées pour les patients suspects d'Ébola		
	• Cette Zone est utilisée uniquement pour les patients suspects d'Ébola		
	• La zone est séparée du bâtiment/bloc principal (zone séparée OU Salle séparée OU tente à l'extérieur)		
	• la zone dispose des éléments suivants pour les cas suspects d'Ébola:<br>a) Gobelets individuels		
	b) pots de latrines individuelles (bassins de lit)		
	c) toilettes ou latrines séparées		
	• La zone est sécurisée (barrière physique)		
	• La zone est bien ventilée		
	• Il y a suffisamment d'espace pour permettre une séparation de 2 mètres entre les patients dans la zone d'isolement		

[4] O = Oui; N = Non; N/A = Non Applicable

STANDARDS DE PERFORMANCE	CRITERES DE VERIFICATION	[5]O, N ou N/A	COMMENTAIRES
**25 Zone pour mettre et enlever l'Équipement de protection Individuel** est aménagée et gérée adéquatement	Observer Si,<br>• L'établissement a des zone désignées pour que le personnel puisse mettre et enlever l'EPI		
	• La zone a tous les éléments d'EPI nécessaires		
	• Un membre de l'équipe est présent dans la zone d'enlèvement des EPI pour superviser ?		
	• Il y a les éléments suivants dans la zone d'enlèvement des EPI :<br>a) Seau rempli de l'eau chloré à 0,5%		
	b) Serviettes jetables		
	C) Un pulvérisateur avec de l'eau chloré à 0,5%		
	d) Poubelle avec un *sac poubelle* en plastique		
	e) Seau rempli de l'eau chloré à 0,05%		
	f) Pédiluve (bain de pieds) rempli de l'eau chloré à 0,5%		
	• Les stations d'EPI sont correctement placées et utilisés aux points de transition entre l'extérieur et les zones à faible risque, et aussi entre les zones à faible risque et les zones à haut risque		
	• Le staff remplace leurs EPI pour chaque patient		

[5] O = Oui; N = Non; N/A = Non Applicable

Procédures de Lavage des mains, port et retrait de l'EPI

STANDARDS DE PERFORMANCE	CRITERES DE VERIFICATION	[6]O, N ou N/A	COMMENTAIRES
**26.** Toutes les salles de procédures disposent **d'équipements et consommable pour le lavage des main**s adéquat pour la PI y compris Ébola	Observer si,<br>• L'eau courante est disponible pour se laver les mains		
	• La bassine est disponible pour le lavage des mains (si oui, est-elle équipée de robinet à pédale ou à main ?)		
	• L'eau pour le lavage des mains avec savon, ou solution chlorée est disponible		
	• La concentration de l'eau chlorée ou un signe qui dit « lavage des mains » est marqué sur le dispositif de lavage des mains		
	• Le savon est disponible pour se laver les mains (indiquer le type : distributeur, morceau, etc.)		
	• Il y a un système de Sèche-mains (indiquer le type: sécheuse, serviettes réutilisables, jetables, l'air ambiant)		
	• Le désinfectant pour les mains à base d'alcool est disponible (indiquer l'emplacement par rapport au point de soins)		
**27.** Le prestataire se **lave les mains avec l'eau et le savon** selon les normes et procédures	Observer si,<br>• Le service assure que de l'eau propre, soit par un robinet, soit dans un récipient avec robinet est disponible		
	• un gobelet, du savon et une serviette individuelle ou des serviettes en papier sont disponibles.		
	• Il y a une poubelle pour jeter les serviettes en papiers.		
	• Le prestataire se lave les mains avant et après chaque procédure et avant et après avoir enlevé les gants		

[6] O = Oui; N = Non; N/A = Non Applicable

STANDARDS DE PERFORMANCE	CRITERES DE VERIFICATION	[7]O, N ou N/A	COMMENTAIRES
	• Le prestataire en se lavant les mains, ouvre le robinet et laisse couler l'eau continuellement ou se sert du gobelet pour verser l'eau régulièrement		
	• Évite les éclaboussures sur les vêtements ou d'autres parties du corps		
	• Applique suffisamment de savon pour couvrir toute la surface des mains.		
	• Frotte les mains paume contre paume avec une motion circulaire.		
	• Frotter la paume de la main droite avec le dos de la main gauche en entrelaçant les doigts.		
	• Frotter la paume de la main gauche avec le dos de la main droite en entrelaçant les doigts		
	• Frotte les paumes l'une contre l'autre en entrelaçant les doigts.		
	• Frotte l'extérieur des doigts contre la paume opposée avec les doigts emboités.		
	• Frotte par rotation le pouce gauche enchâssé dans la paume droite.		
	• Frotte par rotation le pouce droit enchâssé dans la paume gauche.		
	• Frotte par rotation les doigts joints de la main droite dans la paume de la main gauche.		
	• Frotte par rotation les doigts joints de la main gauche dans la paume de la main droite		
	• Rince les mains avec de l'eau.		
	• Arrête de verser l'eau s'il se sert du gobelet.		
	• Sèche les mains avec une serviette individuelle ou un papier absorbant à usage unique		
	• Utilise une serviette pour fermer le robinet.		
	• Jette la serviette dans un récipient à déchets.		
	• Termine la procédure entière entre 40 à 60 secondes.		

[7] O = Oui; N = Non; N/A = Non Applicable

STANDARDS DE PERFORMANCE	CRITERES DE VERIFICATION	O, N ou N/A[8]	COMMENTAIRES
**28** Le prestataire assure **l'hygiène des mains par friction Hydro-alcoolique de manière** correcte. quand c'est indiqué	Vérifier si,<br>• Le service assure la disponibilité d'un produit antiseptique à base d'alcool.		
	• Le prestataire emploi suffisamment de produit dans le creux de la main (3–5 ml) pour couvrir toute la surface		
	• Frotte les mains paume contre paume avec une motion circulaire.		
	• Frotte la paume de la main droite avec le dos de la main gauche en entrelaçant les doigts.		
	• Frotte la paume de la main gauche avec le dos de la main droite en entrelaçant les doigts		
	• Frotte les mains paume contre paume avec les doigts entrelacés.		
	• Frotte l'extérieur des doigts contre la paume opposée avec les doigts		
	• Frotte par rotation le pouce gauche enchâssé dans la paume droite.		
	• Frotte par rotation le pouce droit enchâssé dans la paume gauche.		
	• Frotte par rotation les doigts joints de la main droite dans la paume de la main gauche		
	• Frotte par rotation les doigts joints de la main gauche dans la paume de la main droite		
	• Un fois le désinfectant évaporé, ses mains sont propres et prêtes.		
	• La procédure entière prend fin de 20 30 secondes.		

[8] O = Oui; N = Non; N/A = Non Applicable

STANDARDS DE PERFORMANCE	CRITERES DE VERIFICATION	[9]O, N ou N/A	COMMENTAIRES
**29** Le prestataire **dispose d'un équipement de protection individuelle et le porte** selon les normes et procédures de PI	Observer si,<br>• Une tenue de travail/chirurgicale est disponible.		
	• Tous les articles d'EPI (gants, un tablier en plastique, un masque ou respirateur, des lunettes ou protection faciale, un calot ou un bonnet et des bottes ou chaussures fermées) sont disponibles.		
	• Le prestataire porte l'EPP en fonction de la procédure à exécuter :<br>○ Blouse pour la consultation médicale et gestes non invasifs<br>○ EPP complet pour les activités chirurgicales : gants, un tablier en plastique, un masque, des lunettes ou protection faciale, un calot ou un bonnet, une blouse chirurgicale et des bottes ou chaussures fermées<br>• EPP pour accouchement : gants, un tablier en plastique, un masque, des lunettes ou protection faciale, un calot ou un bonnet et des bottes ou chaussures fermées		
	En présence d'un cas suspect ou confirmer ou pour entre dans une zone d'isolement, vérifier si :<br>• Il y a une zone séparée pour mettre et enlever l'EPI.		
	• Un superviseur/collègue peut aider le prestataire à mettre ou enlever l'EPI, sinon, il peut se servir d'un miroir.		
	• Une fois dans le vestiaire, mettre une tenue de travail		
	• Enfile des bottes en caoutchouc de taille adaptée. Si les bottes en caoutchouc ne sont pas disponibles, porte des chaussures fermées, étanches et résistantes à la perforation et met des couvre-chaussures.		
	• Enfile la tenue ou combinaison Tyvek pour couvrir entièrement le torse du cou aux genoux, des bras à la fin des poignets, et l'enroule autour de son dos		
	• Fixe la tenue à l'arrière du cou et de la taille en veillant à bien recouvrir les poignets si nécessaire.		

[9] O = Oui; N = Non; N/A = Non Applicable

	• Met un masque médical ou un respirateur N-95.		
	• Si il porte un masque, pince au niveau du nez pour assurer que le masque est correctement positionné.		

STANDARDS DE PERFORMANCE	CRITERES DE VERIFICATION	O, N ou N/A[10]	COMMENTAIRES
	• Si il porte un respirateur N-95, l'ajuste pour qu'il épouse les contours du nez et de la bouche :<br>a. Expire profondément. Si une pression positive s'accumule, il n'y a pas de fuite. Si la pression n'augmente pas, règle le respirateur.<br>b. Inspire profondément. S'il n'y a pas de fuite, le respirateur s'accroche au visage. Sinon, ajuste le masque.		
	• Fixe les attaches ou bandes élastiques au sommet de la tête et à la base du cou.		
	• Met des lunettes de protection ou un écran facial.		
	• Met un calot/un bonnet, et s'assure que la tête est entièrement couverte		
	• Porte une paire de gants.		
	• Se sert de ruban adhésif pour assurer que les gants sont connectés à la tenue et pour sceller tout espace.		
	• Met une deuxième paire de gants		
	• Enfile un tablier en plastique.		
	• NE TOUCHE OU N'AJUSTE pas L'EPI. Demande à un superviseur ou collègue de l'aider.		
	• Demande au superviseur/collègue d'utiliser la liste de vérification pour s'assurer que l'EPI est au complet et ajusté correctement. Ajuste correctement si nécessaire. Si la personne se sert d'un miroir, vérifie que tous les articles de l'EPI sont en place et rectifie si nécessaire. **Avant d'entrer dans la salle d'isolement ou dans la zone de soins aux patients.**		

[10] O = Oui; N = Non; N/A = Non Applicable

STANDARDS DE PERFORMANCE	CRITERES DE VERIFICATION	O, N ou N/A[11]	COMMENTAIRES
**30** Le prestataire **retire son équipement de protection individuelle** selon les normes et procédures de PI	Observer si,<br>• Le prestataire s'assure qu'un superviseur ou collègue est disponible pour l'aider.		
	• Ce collègue porte l'EPI ou se tient à au moins 2 mètres de lui		
	• Des récipients contenant une solution de chlore à 0,5% sont disponibles pour la décontamination de l'EPI après le retrait.		
	• Un sac à déchets est en place pour y déposer en sécurité l'EPI utilisé		
	• Pulvérise le devant et l'arrière de l'EPI avec une solution de chlore à 0,5%.		
	• Enlève le tablier en plastique et le met dans le sac à déchet que le collègue tient. Si l'EPI doit être réutilisé, le mettre dans un récipient avec du désinfectant.		
	• Trempe les mains gantées dans une solution de chlore à 0,5%.		
	• Enlève les chaussures avant d'enlever les gants.		
	• Trempe les mains gantées dans une solution de chlore à 0,5%.		
	• Retire la deuxième paire de gants et mettre dans un sac à déchets.		
	• Retire le ruban adhésif de la première paire de gants.		
	• Enlève la blouse, ou s'il porte une combinaison Tyvek, défait la fermeture, enlève la capuche, et l'enroule vers l'intérieur		
	• Trempe les mains gantées dans une solution de chlore à 0,5%.		
	• Enlève le calot ou le bonnet.		
	• Trempe les mains gantées dans une solution de chlore à 0,5%.		

[11] O = Oui; N = Non; N/A = Non Applicable

STANDARDS DE PERFORMANCE	CRITERES DE VERIFICATION	O, N ou N/A[12]	COMMENTAIRES
	• Enlève les lunettes de protection ou l'écran facial de l'arrière à l'avant.		
	• Trempe les mains gantées dans une solution de chlore à 0,5%.		
	• Retire le masque en le saisissant par l'arrière.		
	• Trempe les mains gantées dans une solution de chlore à 0,5%.		
	• Retire les gants et les jette dans un sac à déchets.		
	• Désinfecte les bottes/chaussures en pulvérisant la partie externe avec une solution de chlore à 0,5% avant de quitter la zone contaminée.		
31. Dispositions appropriées sont prises au niveau de la Morgue pour la PI	Observer si les prestataires de la morgue :<br>• Utilisent l'EPI (bottes, Gants de ménage, lunettes, Tablier,) au cours de la manipulation des corps<br>• A la réception des corps, une note qui précise la cause probable du décès est jointe.<br>• Les corps des personnes suspects d'Ébola sont traités dans un endroit séparé et sont enterrés par des prestataires formés pour cette activité	________<br>________<br>________	
32. La formation sanitaire dispose de matériels et équipements pour l'EPP en en quantité suffisante pour au moins un mois	Vérifier lors de la visite la disponibilité de :<br>• Tabliers jetables / Blouses<br>• Tabliers réutilisables / Blouses<br>• Masque pour le visage<br>• Gants (stérile, chirurgical, GYN)<br>• Pulvérisateurs<br>• Lunettes<br>• Bottes en caoutchouc<br>• Alcools (isopropylique)<br>• Ecran facial /visière<br>• Désinfectant pour les mains<br>• Pulvérisateurs	______<br>______<br>______<br>______<br>______<br>______<br>______<br>______<br>______<br>______<br>______	

[12] O = Oui; N = Non; N/A = Non Applicable

	• Seaux avec robinet<br>• Bassins<br>• Eau de javel, de 0,5%<br>• Thermomètres (Thermo-Scan ou Numériques)<br>• Boîtes de sécurité	______<br>______<br>______<br>______<br>______	

Nombre total de standards	**33**
Nombres total observés	
Nombre total atteints	
**Performance :**	**%**

NB : il y a 33 standards mais retenons car il y a 9 et 9a.
